# Wheel-Running Behavior Is Negatively Impacted by Zinc Administration in a Novel Dual Transgenic Mouse Model of AD

**DOI:** 10.3389/fnins.2020.00854

**Published:** 2020-08-14

**Authors:** Stephen L. P. Lippi, Peter A. Kakalec, Meghann L. Smith, Jane M. Flinn

**Affiliations:** ^1^Angelo State University, San Angelo, TX, United States; ^2^George Mason University, Fairfax, VA, United States

**Keywords:** zinc, circadian activity, Alzheimer’s disease, mouse model, sex, age

## Abstract

Alzheimer’s disease (AD) is a neurocognitive disorder that impacts both the brain and behavior. Metal ions, including zinc (Zn), have been seen to play an important role in AD-related pathology. In this study, we show alterations in wheel-running behavior both early and late in disease progression in a novel dual Tg mouse model of AD. This mouse includes both amyloid and tau pathology through its cross with the J20 (hAPP) and P301L (Tau) parentage. Animals were given either lab water or water that had been supplemented with 10 ppm Zn. Wheel running was assessed through individually housing mice and measuring wheel-running activity in both the light and dark cycles. Dual Tg mice showed significantly less activity in the first part of the dark cycle than WT mice at both 3.5 and 7 months of age (*p* < 0.05). Dual Tg mice given Zn water showed less activity compared to dual Tg mice on lab water, tau mice on Zn water, or WT mice given either lab or Zn water (*p* < 0.05) at 7 months. Female mice in this study consistently showed higher activity compared to male mice in all groups whereas Zn led to reduced activity. Daily activity rhythm was altered in both the tau and dual Tg mice, and Zn impacted this alteration through effects on amyloid, tau, and through circadian pathways.

## Introduction

Alzheimer’s disease (AD) is a neurodegenerative condition that involves accumulations of harmful proteins, including amyloid plaques and tau tangles. The prevalence of AD is significant: approximately 5.8 million Americans are diagnosed today and, with populations living longer, these statistics are projected to increase substantially by the year 2025 (Alzheimer’s Association, 2019). Apart from progressive neuronal pathology, AD leads to many behavioral problems, the most characteristic of which is loss of memory. Apart from substantial memory deficits, which worsen as the disease progresses, there are other behaviors that are impaired. One which causes significant problems for caregivers is a disturbance in the circadian rhythms (CR) of AD patients; this is one of the major reasons for institutionalization (Bianchetti et al., 1995; Coogan et al., 2013; Duncan and Zee, 2013; Holth et al., 2017).

Previous research and drug development has focused on amyloid (Cummings et al., 2018) but tau is becoming increasingly important to examine when studying AD. Tau pathology is considered to correlate better with cognitive diminishment than amyloid based on its distribution and progression across the brain (Braak and Braak, 1991) and no amyloid-targeting therapies in the drug-development pipeline have been successful to date (Kametani and Hasegawa, 2018).

Disturbances in the sleep-wake cycle are noted in AD patients (Musiek et al., 2015) and have been seen in numerous mouse models of AD. These disruptions have been seen in not only the 3xTgAD (Sterniczuk et al., 2010) and 5xFAD mouse models (Sethi et al., 2015) but also in solely amyloid (Wisor et al., 2005) and tau (Craven et al., 2018) mice. As a non-cognitive measure, disturbances in circadian activity can be an early indicator of and a precursor to upcoming dementia (Tranah et al., 2011; Leng et al., 2019).

One factor that may be mediating AD behavioral changes is the bio-metal, zinc (Zn). Zinc, Iron (Fe), and Copper (Cu) are all found in the plaques associated with AD (Lovell et al., 1998; Suh et al., 2000) and these metals can affect both behavior and the progression of the disease (Linkous et al., 2009; Railey et al., 2011; Bush, 2013; Adlard and Bush, 2018; Craven et al., 2018). In AD, the homeostasis of these crucial metals is disturbed and can cause exacerbations in neuronal pathology. Zinc’s interaction with amyloid leads to decreased amounts of Zn availability; this can impact neuronal signaling as less Zn is available for co-release with glutamate in synaptic transmissions. Both amyloid (Aβ) and neurofibrillary tau interact with Zn (Mo et al., 2009; Barnham and Bush, 2014; Huang et al., 2014). Zinc can affect both kinase activity (Boom et al., 2009) and phosphatase activity (Xiong et al., 2013) which leads to alterations in tau’s structure and its propensity to aggregate.

*In vitro* studies are valuable in assessing the impact of Zn on tau and Aβ, however, changes in the levels of one metal can affect the levels of others *in vivo* (Maret and Sandstead, 2006). The use of animal models for AD allows for more translational findings and the ability to see the impact that Zn has on behavior in conjunction with AD pathology. Excess zinc causes impairments in many behaviors, including CR, in mice modeling AD which contain either human amyloid or human tau (Graybeal et al., 2015; Boggs et al., 2017; Craven et al., 2018). We have now examined changes in daily activity rhythm in a mouse model of AD containing *both* human amyloid and human tau (Lippi et al., 2018) and examined the effect of zinc administration in these mice. Mice were examined at both 3.5 and 7 months; sex differences in activity were also examined. We report here the results of a study of daily activity rhythm in a mouse model of AD that contains both human amyloid and human tau. Genotype (Tg v. WT) had a significant effect; in addition, zinc administration, sex, and age all had effects on daily activity rhythm in the Tg mice, but not on the Wt mice, with zinc having the greatest effect on the dual Tg mice.

## Materials and Methods

### Animals

Dual Tg mice (those mice arising from the cross between the J20 and rTg4510 mouse models, Jax) have been previously characterized by Lippi et al. (2018) and were used to assess wheel-running activity compared to those mice containing P301L tau under the tetracycline transactivator system, and mice containing no AD mutations (wildtype, WT). Male mice used in the breeding were those harboring the SWE and IND APP mutations (J20 – B6.Cg-Zbtb20^Tg(PDGFB–APPSwInd)20Lms^/2Mmjax); these mice are on a B6 background and are hemizygous. Female mice used in the breeding were those with the P301L tau mutation under control of the tetracycline transactivator (rTg4510 – Tg(Camk2a-tTA)1Mmay *Fgf14*^Tg(tetO–MAPT*P301L)4510Kha^/J); the female mice were on a mixed FVBxC57BL/6 background, as they are maintained on this background at the Jackson Lab. The rTg4510 mice used were hemizygous for Tg(Camk2a-tTA)1Mmay and hemizygous for Fgf14^Tg(tetO–MAPT*P301L)4510Kha^. These breeding mice were paired and offspring were genotyped (Transnetyx, Inc.). Offspring used in this study were of the F1 generation. “Wildtype” mice used and discussed in this paper are those mice that were genotyped and had no presence of any of the used mutations. Mice were assessed at both 3.5 and 7 months, to assess early and later stage changes in pathology.

Mice were housed based on sex and genotype. Additionally, mice were housed with littermates. Each cage (Animal Care Systems) contained a running wheel and an igloo with food and water given *ad libitum.* Rat cages were used, as opposed to mouse cages, to allow enrichment devices to fit while having multiple mice in a cage. The light cycle was maintained on a 12-h light/12-h dark cycle consistent with animal colony housing.

All procedures done involving animals in this project were approved by the George Mason University IACUC.

### Zinc Water

Mice were given either standard laboratory water or water with 10 ppm Zn added. Zinc water (ZnCO3) was prepared with a 10,000 ppm solution dissolved in 5% nitric acid and buffered with sodium carbonate to reach neutral pH (∼7). Samples were sent to the United States Geological Survey (USGS) (Reston, VA, United States) to ensure accurate Zn levels throughout the duration of the experiment. This formulation of 10 ppm Zn has been done in prior studies assessing Zn’s impact on Tg mice from our lab (Linkous et al., 2009; Flinn et al., 2014; Craven et al., 2018). Mice in the Zn water condition began receiving Zn water at 8 weeks of age and continued throughout the length of the study. A breakdown of animals by sex, genotype, and water condition can be seen in Table 1. This table represents the group Ns at both time periods (the same mice that were assessed at 3.5 months are the same as those analyzed at 7 months).

**TABLE 1:** Study sample size.

	Dual Transgenic (*n* = 18)		Tau (*n* = 23)		Wildtype (*n* = 20)	
	Male	Female	Male	Female	Male	Female
Lab Water	4	4	4	7	5	5
Zinc Water	5	5	5	7	6	4
***N* = 61**						

### Wheel Running (Daily Activity Rhythm Measurement)

Mice in the current experiment underwent a battery of behavioral tests, including those to measure learning and memory (Barnes Maze), anxiety [open field test and elevated zero maze (EZM)], depression (forced swim test), and activities of daily living (burrowing and nesting) (Lippi et al., 2018). After burrowing and nesting behavior were assessed, and prior to forced swim activity data collection, mice were tested for daily wheel-running activity.

Each mouse was singly housed in a circadian rhythm (CR) running wheel cage (Coulbourne Instruments). Cages consisted of one voluntary running wheel, corncob bedding (to avoid entanglement with the wheel), and a hopper for food and water (whether lab or Zn-supplemented). Actiview Biological Rhythm Analysis software (Minimitter Co.) was used to record the number of wheel rotations for each minute.

Wheel-running activity for respective CR cages was recorded for a total of 9 days, with the first (cage set-up) and last (cage take-down) days excluded from analysis. Total wheel rotations were averaged across the median 7 days to generate a mean number of wheel rotations for each hour of the 24-h cycle.

### Statistical Analysis

Wheel-running data from the CR cages were summed for each hour and then analyzed by days. The hourly data for each day were averaged to give an average for each hour. This data was then split into separate 12-h light (ZT0-12) and dark (ZT12-23) cycles for analysis. Light and dark cycle data were analyzed using a 3 × 12 repeated measures ANOVA with factors of genotype, water type, and sex by hour. Significant results were followed-up with an ANOVA to see at what times significant differences were present, using three or more consecutive significantly different time intervals as a post-hoc. All data were analyzed using *p* < 0.05 as the cutoff for significance, and *p* < 0.10 to indicate a trend.

## Results

Animals were tested twice, once at age 3.5 months and again at 7 months. We were interested in assessing pathology early and late in disease progression, so analyses for each testing session/age were performed separately. Mauchly’s Test of Sphericity was significant for all analyses so a Greenhouse-Geisser correction was used. Analysis of light cycle data revealed a significant hour × genotype effect at 3.5 months (*p* < 0.05), however, post-hoc analysis was unable to reveal an individual effect or time range for significance, and no differences were observed at 7 months. Therefore, this analysis focuses solely on dark cycle data. Data were analyzed in the following order: effect of genotype, effect of water, and effect of sex. Finally, all data were considered together.

### Effect of Genotype

**FIGURE 1:**
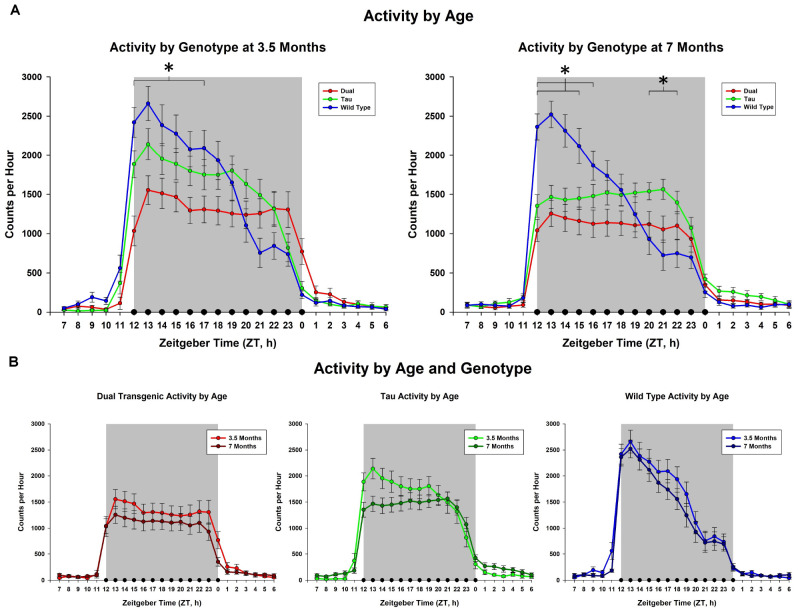
Comparison of dark cycle activity by **(A)** Genotype by age and **(B)** age by genotype. Wildtype animals showed significantly higher total dark cycle activity than dual Tg animals at 7 months; hourly differences showed dual Tg animals were significantly less active than wildtypes early in the dark cycle, ZT12-17 at 3.5 months and ZT12-16 at 7 months. At 7 months tau animals were significantly less active than WT mice early in the dark cycle, ZT12-15, and significantly more active than WT mice later, ZT21-23 (^∗^*p* < 0.05).

This analysis collapsed animals across sex and water. No overall effect was seen for the 3.5 month mice; however, effects of hour × genotype were seen at both 3.5 months [*F*(5.86,143.48) = 9.771, *p* < 0.001, Figure 1A], and 7 months [*F*(5.789,141.66) = 14.331, *p* < 0.001, Figure 1A]. Follow-up analysis of the hour × genotype effect showed the dual transgenic animals were less active than the wild type animals at hours ZT12-17 at 3.5 months, (consecutive *p* < 0.05) and ZT 12-16 at 7 months (consecutive *p* < 0.05) (Figure 1A). Additionally, at 7 months tau animals were less active than wild type animals at hours ZT12-15 (consecutive *p* < 0.05), and more active at hours ZT21-23 (consecutive *p* < 0.05). An effect of genotype on total dark cycle activity was present at 7 months [*F*(2,49) = 3.18, *p* = 0.05, Figure 1A], Follow-up analysis of the effect of genotype on total dark cycle activity showed that dual transgenic animals were less active than wild type animals at 7 months (*p* < 0.05).

### Effect of Drinking Water

**FIGURE 2:**
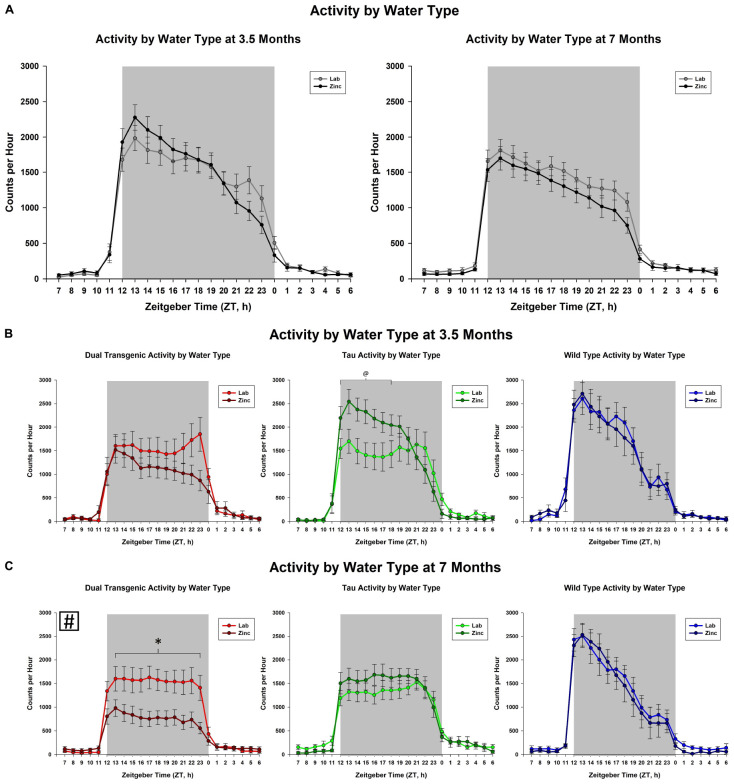
Comparison of dark cycle activity by **(A)** water type by age, **(B)** water type by genotype at 3.5 months, and **(C)** water type by genotype at 7 months. Tau animals exclusively showed a trend for increased activity from ZT 13-15 (^#^) when administered Zn at 3.5 months. Dual Tg animals on Zn were significantly less active than all other groups except tau lab at 7 months (^∗^*p* < 0.05, ^@^*p* < 0.10, boxed ^#^ indicates a change summed across dark cycle hours).

The effect of adding zinc to the drinking water was then examined. This analysis collapsed animals across sex. An effect of hour × water was observed at 3.5 months [*F*(2.93,143.48) = 4.29, *p* < 0.01, Figure 2A], but not at 7 months. We observed a trend for an effect of hour × water × genotype at 3.5 months [*F*(5.86,143.48) = 1.89, *p* < 0.1, Figure 2B]. Follow-up analysis of the hour × water × genotype effect at 3.5 months showed that zinc increased activity only in the tau animals early in the dark cycle from hours ZT 13-15, (all consecutive *p* < 0.05). Although there was not an effect of adding Zn at 7 months, there was an interaction between water × genotype which affected total dark cycle activity at 7 months [*F*(2,49) = 4.10), *p* < 0.05, Figure 2C] (Follow-up analysis of the interaction at 7 months showed that the dual transgenic animals on zinc water were less active than dual transgenic lab, tau zinc, WT lab, and WT zinc animals), i.e., significantly less active than all other groups except for tau lab.

### Effect of Sex

**FIGURE 3:**
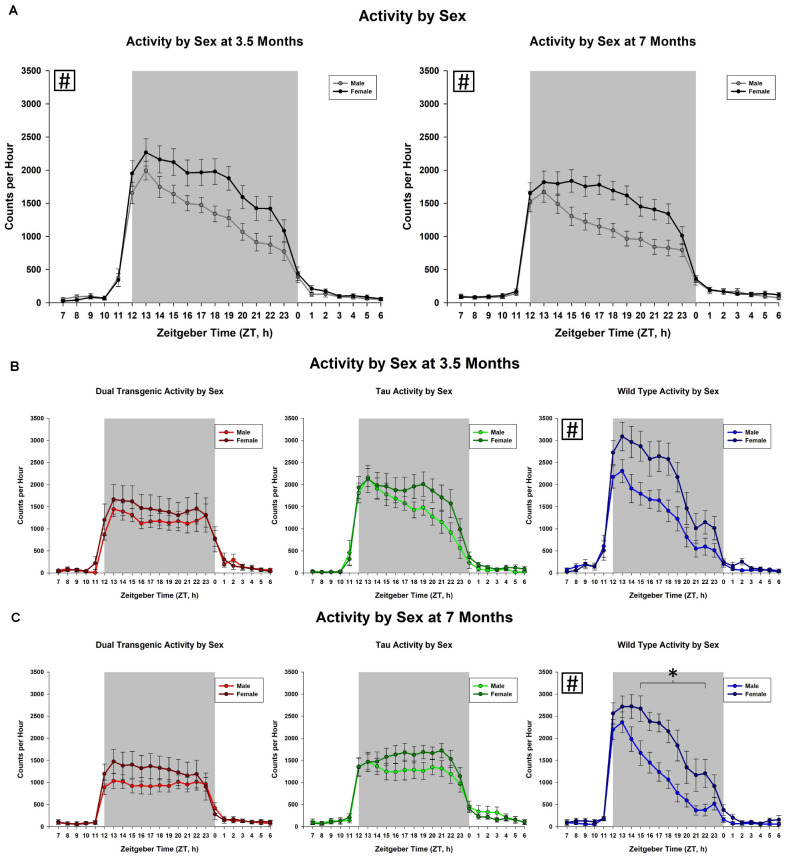
Comparison of dark cycle activity by **(A)** sex by age, **(B)** sex by genotype at 3.5 months, and **(C)** sex by genotype at 7 months. Female animals showed significantly higher dark cycle activity compared to males at both 3.5 and 7 months **(A)**. At 7 months, this increase was present in hours ZT 15-22 (all time intervals, *p* < 0.05). Female wild type animals were significantly more active than wild type males at both 3.5 and 7 months (^∗^*p* < 0.05, boxed ^#^ indicates a significant effect summed across dark cycle hours).

We then examined the effect of the sex of the mice. This analysis collapsed animals across water type. We observed an effect on total dark cycle activity at both 3.5 [*F*(1,49) = 6.29], *p* < 0.05 and 7 [*F*(1,49) = 11.50, *p* < 0.001, Figure 3A] months. Female animals were more active in the dark cycle than the males. A trend for an effect of hour × sex was present only at 7 months [*F*(2.89,141.66) = 2.18, *p* < 0.1, Figure 3A]. The hour × sex effect at 7 months showed female animals were more active from ZT 15-22, from the mid to late dark cycle (all consecutive *p* < 0.05). There was a significant effect of sex in wild type animals on total dark cycle activity levels at both 3.5 [*F*(1, 18) = 7.15, *p* < 0.05] and 7 [*F*(1, 18) = 9.5, *p* < 0.05] months.

### Genotype, Water, and Sex Together

**FIGURE 4:**
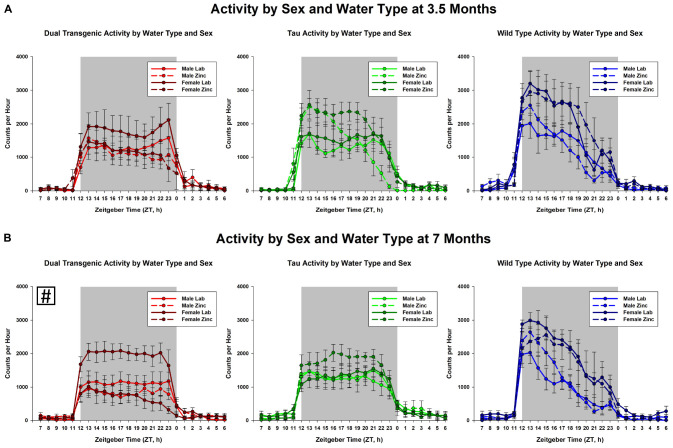
Comparison dark cycle activity by **(A)** sex by water type by genotype at 3.5 months, and **(B)** sex by water type by genotype at 7 months. At 7 months, dual Tg females on lab water were significantly more active than either sex on Zn water, *p* < 0.05 (boxed ^#^ indicates a significant effect summed across dark cycle hours).

The effect of water type by sex on dark cycle activity was broken down by genotype and age (Figure 4). The data show that in general, females were more active than males and zinc reduced activity. This was most notable in the 7 month dual transgenic mice, where the lab females had significantly higher activity than either sex on zinc (both *p* < 0.05). but did not differ significantly from males on lab water.

## Discussion

We have shown that a novel mouse model, previously shown to exhibit deficits in both cognitive (Barnes maze) and non-cognitive (burrowing and nesting) behaviors (Lippi et al., 2018), exhibited disturbances in wheel-running activity both early and late in development of AD pathology. Analysis showed that the activity of the Wt mice did not vary with age, zinc administration, or sex; however, these variables did affect both the tau and dual Tg mice, with the dual Tg mice being the most affected, particularly with respect to zinc administration.

Activity during the dark cycle typically starts at a high rate and falls off as the night progresses in Wt mice (Boggs et al., 2017; Logan et al., 2018), and this pattern was seen here in the Wt mice at both 3.5 and 7 months. The pattern was similar in the tau mice at 3.5 months, but began to differ from the Wt mice at 7 months, where the level of activity in the tau mice showed less variation across the night, as shown in Figure 1. The dual Tg mice had an atypical pattern at both 3.5 and 7 months, with a consistent level of activity across the dark cycle at both ages. Activity in AD mice in our sample was lower than that of the WT mice in the early part of the night cycle at both 3.5 months and 7 months. This lowered activity in AD mice is in agreement with Wu et al. (2018) who used the 3xTg-AD mouse, containing mutations in amyloid, tau, and the PS1 gene, and found that AD mice had lower total daily activity in running compared to WT mice.

We noted significant differences in the *pattern* of activity across the night in the three groups at both 3.5 and 7 months (see Figure 1); the greatest difference was between the WT and dual Tg animals. Compared to WT mice, who at both testing ages showed similar characteristic patterns of falling activity across the dark cycle, dual Tg mice had relatively constant activity at both ages.

This mouse model may be suitable for future daily activity rhythm analysis since wheel-running activity was disrupted early on; others have assessed wheel running in AD mice at later ages (Duncan et al., 2012; Boggs et al., 2017; Craven et al., 2018; Wu et al., 2018). Further analysis showed that the activity of the Wt mice did not vary significantly with age, zinc administration, or sex; however, these variables did affect both the tau and dual Tg mice, with the dual Tg mice being the most affected, particularly with respect to zinc administration. There was an overall effect of sex, with females showing greater activity than the males. The greatest differences in activity due to Zn administration were seen in the dual Tg mice at 7 months. These results are discussed in more detail below.

### Alzheimer’s Disease Pathology and Circadian Activity in Mice

Previous studies have shown that progression of amyloid and tau pathology can lead to disruptions in circadian rhythm activity in mice. In C57BL/6J mice with no AD mutations, administration of the Aβ31-35 fragment into the hippocampus led to an increase in free-running period and changes in Per1 and 2 in both the suprachiasmatic nucleus (SCN) and hippocampus in constant darkness (Wang et al., 2016). Per1 and Per 2 were shown to be lower in mice receiving the fragment in both the SCN and the hippocampus.

Transgenic mice harboring mutations in AD genes have also shown disruptions in wheel-running activity. Sterniczuk et al. (2010) assessed circadian wheel-running activity in the 3xTgAD mouse model. Their results corroborate the findings in the present study: both young and old 3xTgAD mice exhibited less nighttime activity compared to WT/control mice. Tau mice harboring the P301L mutation had delayed activity onset compared to WT mice (Craven et al., 2018) and this delay was exacerbated by Zn administration. P301L tau mice, that have tau expression regulated through CaMKIIa promotion, had delayed activity onset during the evening, with P301L tau mice receiving Zn water showing a further delay in activity onset compared to those receiving lab water (Craven et al., 2018). The present study showed that Zn led to alterations early on in the P301L tau animals, with Zn unexpectedly increasing the activity early in the dark cycle (Figure 2B).

A late-onset model of AD explored by Boggs et al. (2017) showed that, compared to C57 controls, APP/E4 (transgenic) mice did not exhibit a typical decline in activity throughout the night. Instead, similar to the dual Tg mice here, there was only a slight decrease, with lower activity compared to control mice during the beginning of the dark cycle and higher levels at the end of the dark cycle at both young (pre-plaque) and old (post-plaque) ages.

### The Role of Zinc in Daily Rhythm Activity

Increasing zinc through the drinking water had no effect on the daily rhythm activity in Wt mice in this study, which is consistent with results from other studies (Craven et al., 2018; Moshirpour et al., 2020). However, there was a significant effect for the dual Tg mice, where activity decreased, and a smaller effect for the tau mice, where activity increased.

As shown in Figure 2C, the dual Tg mice given Zn water had lower activity than all groups except tau lab mice at 7 months, but this was not seen at 3.5 months. They also had the lowest activity of all groups studied.

Zinc is found in a number of regions in the circadian rhythm circuit (Moshirpour et al., 2020) including the intergeniculate leaflet (IGL) of the thalamus and the melanopsin-containing retinal ganglion cells which feed into the suprachiasmatic nucleus and the IGL. The data indicate that Zn interacts with amyloid and or tau in some region of the circuit, as yet unknown. Overall, what we present here is that there was a much stronger effect at 7 months; this may be due to the effect of increased levels of tau and/or amyloid interacting with Zn ions.

Zinc does not affect daily activity rhythms in wildtype mice at 7 months, thus indicating that the reduced activity seen in the dual Tg mice is due to an interaction with Zn and amyloid or tau. The changes are not due to a loss of activity in general in the 7 month dual Tg mice since there were no significant differences in distance traveled in the open field test between dual and tau mice at this age (Lippi et al., 2018).

### The Role of Zinc in Sleep

Zinc’s impact on sleep has been investigated in both humans and animals. Cherasse et al. (2015) showed that Zn, in the form of a Zn-containing yeast extract, led to decreases in locomotor activity in a dose-dependent manner, but only when given at the beginning of the dark cycle. This decrease in activity was accompanied by an increase in NREM sleep. Zinc concentrations in this range 1–160 mg/kg were administered; there was a dose-dependent effect. The 40 mg led to a drop of ∼16% but the maximum effect was seen at 80 mg/kg where the reduction was over 50% and similar to that at 160 mg. Additionally, yeast extract led to an increase in NREM sleep in mice given either 40 mg/kg or 80 mg/kg Zn compared to vehicle; in both cases, the NREM sleep increased in the first hour. This dose is higher than what was used in the current study; the lower concentrations of Zn-enriched yeast are more similar to the present study’s make up of Zn administration. These lower doses were not significantly different from vehicle administration given by Cherasse et al. (2015). Therefore, the lower activity seen in the dual Tg mice given Zn could be a product of Zn administration and interaction with AD pathology, rather than the Zn itself.

In a review on the way that dietary Zn can impact sleep, Cherasse and Urade (2017) discussed how Zn administration can impact sleep quality and amount in mice and humans. As evidence for the effects of Zn supplementation in humans, latency for sleep-onset and sleep efficiency were measured after being given either oysters, a food rich in Zn, or supplements aimed to impact Zn absorption. Specifically, the group eating oysters were shown to have significantly improved sleep efficiency and lower sleep-onset latency compared to the placebo group (Saito et al., 2016). This study showed that Zn has the ability to improve sleep after being ingested for several months. Baradari et al. (2018) also found that zinc improved sleep quality and sleep latency in nurses; the dose used was 220 mg. However, ingestion of too much Zn may be problematic in AD patients since administration of Zn could possibly lead to enhanced AD pathology as discussed below.

### The Effect of Zinc on Alzheimer’s Disease as a Function of Age

The impairments in daily activity rhythms were seen at 3.5 months in both mice raised on lab water and Zn enhanced water, with the Zn effects increasing significantly at 7 months in the dual Tg mice. This is a different pattern than that seen with other measures in these mice, such as the Barnes maze, nesting, or burrowing (Lippi et al., 2018). In these cases, the impairment of mice on lab water was already severe at 3.5 months and Zn had no further effect. The fact that Zn produced a stronger effect on daily activity rhythms at 7 months, with the dual Tg mice showing such low levels of activity in both male and female mice, suggests that there may be a different mechanism at work, potentially involving the eye.

The relationship between Zn and AD is complex. Zinc can interact with both amyloid and tau as these species tightly bind the metal, leading to higher propensities of Aβ aggregation and tau fibrillization (Mo et al., 2009; Miller et al., 2010). One theory of AD (Bush, 2008) suggests that Zn and Cu both bind with amyloid in the synaptic cleft; thus, the levels of both could be reduced. Lippi et al. (2018) confirmed this reduction in Zn through use of Zinpyr-1 fluorescence in the hippocampus, demonstrating that dual Tg mice had less free Zn than wildtype mice. Since Zn competes with Cu for entry into the body, increased Zn in the drinking water could enhance the Cu deficiency, and one study shows that a Zn-related spatial memory deficit in mice with APP is remediated by adding Cu (Railey et al., 2011). However, Zn also interacts with tau, and Craven et al. (2018) and Lippi et al. (2018) both show that Zn can increase hyperphosphorylation in tau mice. Additionally, Lippi et al. (2018) showed that the dual Tg mice had less free Zn in the hippocampus compared to wildtype mice; this is hypothesized to be due to increased binding with amyloid and tau pathology present within the hippocampus of these mice. Thus, Zn can have a negative effect in AD due to its interaction with both tau and APP.

### Measuring Zinc in the Brain

As indicated above, we did find reduced free Zn in the hippocampus of the dual Tg mice (Lippi et al., 2018). Although Zn measurements are not presented in this paper, in other research, our method of Zn administration (10 ppm ZnCO3) was shown to increase free Zn fluorescence in the hippocampus of wildtype mice (Lippi et al., 2019).

In order to measure total Zn, both bound and free, researchers can use a number of methods such as ICPMS and X-ray synchrotron fluorescence (Flinn et al., 2005; Neely et al., 2019). However, levels of free Zn are notoriously hard to measure. Lippi et al. (2018) showed that dual Tg mice had significantly less free Zn in the hippocampus compared to wildtype mice, using Zinpyr-1 (a probe measuring free Zn fluorescence). This reduction in free Zn is hypothesized to be due to interactions with AD proteins that can bind Zn ions directly. Zinpyr-1 has also been used by Lippi et al. (2019) to show that the dose of Zn water given to these animals (10 ppm ZnCO3) leads to greater amounts of free Zn fluorescence (an indicator of more free Zn) in the hippocampus of wildtype mice. Despite use of these Zinpyr probes or X-ray synchrotron fluorescence, levels of free Zn are notoriously hard to measure. Maret (2015) states that although probes and sensors can provide qualitative differences in free Zn ion concentrations, when researchers seek to establish quantitative differences, there needs to be consideration for the assumptions underlying the use of probes and sensors. Additionally, changes in free Zn ions tend to be compared using ratios.

### Sex Differences

An important factor to consider when measuring behavioral changes in mouse models of AD is biological sex of the animal subjects. Sex differences were noted at both testing ages. At 3.5 months, overall, female animals showed significantly more dark cycle activity than males and at 7 months they were significantly more active than males from the mid to late dark cycle (Figure 3A). These differences were significant in the Wt mice and similar patterns of activity were seen amongst the other conditions (dual Tg and tau), although not statistically significant (Figures 3B,C). Wu et al. (2018) showed similar results, in that the total daily activity in female mice was greater than that of male mice, regardless of genotype (WT v. 3xTg-AD). While Wu et al. (2018) tested at 6 months of age, we saw these differences at an early testing age; at 3.5 months; however, the differences were less in the transgenic mice than in the Wt mice.

While research shows that women experience AD more than men (Viña and Lloret, 2010; Li and Singh, 2014), as compared to mild cognitive impairment (MCI) (Mielke et al., 2014), studies conducted in non-human models still often only use male mice. However, a number of studies included both sexes and showed different circadian rhythms in the sexes. Mouse models assessing circadian rhythms and sleep solely in male mice, have shown that age can impact sleep and wheel running activity in non-Tg inbred mouse strains (Valentinuzzi et al., 1997; Hasan et al., 2012). The 3xTg-AD mouse model showed altered circadian locomotor rhythms (Wu et al., 2018) with female mice showing less disruptions compared to males. Female 3xTgAD mice have also been found to be less active than non-Tg mice during the evening (lights-off) period (Sterniczuk et al., 2010). By including sex as a factor, research with AD mouse models can be more inclusive of the actual population as well as highlight differences between males and females which may lead to a better understanding of why females are different than males with regards to AD.

### Interaction Between Sex, Zinc, and Genotype

The dependence of activity on sex, water type, and genotype is shown for 3.5 and 7 months in Figure 4. The greatest effect was seen for the dual Tg mice at 7 months, where both the male and female mice on Zn had significantly lower activity than the females on lab water.

### Background Strains and Their Impacts on Circadian Activity

Daily activity rhythms, as well as open field activity and body temperature have been shown to be impacted by background strains of mice (Connolly and Lynch, 1981). Circadian disruption (leaving the lights on constantly) has been shown to lead to behavioral differences in male C57BL6/N and C57BL6/J mice (Capri et al., 2019). Earlier research has also shown that control strains of mice also differ in stability of circadian rhythms when exposed to constant darkness (Ebihara et al., 1978). These differences in activity, temperature, and circadian rhythms could impact research involving groups of mice not all bred from the same set of breeder parents or in projects involving ordering different control animals than a mixed background breeding scheme allows.

The mixed strain characteristic of the F1 mice used in this study is a component to address. The FVB/N and C57BL6/J strains have been compared in the literature as it relates to circadian wheel activity (Pugh et al., 2004). They found that, compared to C57BL6/J mice, FVB/N mice had greater activity in the light phase and never reached the same level of activity during the dark period. C57BL6J mice were also shown to entrain well to a 12:12 light/dark cycle compared to the FVB/N mice. It is important to note, however, that only male mice were used in the circadian wheel activity analysis and that in the current project, the F1 generation was of a mixed background. Wildtype mice (those genotyped as having no mutations in hAPP or tau, and those not including the promoter to express tau) were littermates and produced through the breeding process, helping to control for any additional background differences that may have impacted behavioral differences.

One possible confound is that the genetic makeup of the rTg4510 mouse contains a deletion involving a portion of Vipr2, a gene that plays a role in circadian activity and is found in the suprachiasmatic nucleus (Harmar et al., 2002). Harmar et al. (2002) have shown that the overall activity of Vipr2-/- mice is significantly less than that of Wt animals; thus affecting Vipr2 could have caused a loss in activity in the tau mice. Vipr2+/- mice have a reduced ability to bind their selective ligands, which indicates a gene-dose effect (Harmar et al., 2002); these mice would be similar to those mice used in the current study containing the CaMKIIa-tTA transgene. The tau mice did show a lower rate of activity overall than the WT mice at 3.5 months (Figure 1A), although this difference was not significant. However, at 7 months tau animals had a different overall pattern of activity, being significantly less active than WT animals at ZT 12-15 and more active than WT mice at ZT 21-23 (Figure 1B). In contrast, at 3.5 months, the dual Tg mice were significantly less active than the WT mice in ZT 12-17, and at 7 months they were less active than the WT mice at ZT 12-16. As AD pathology worsened, their activity decreased; this behavior in the dual Tg mice indicates an effect above and beyond that of the tau transgene and its effect on the Vipr2 gene. Additionally, Boggs et al. (2017) found that mice with both APP and E4 had a reduced level of activity, again showing an impairment due to AD pathology. Future studies assessing daily rhythm activity in the rTg4510 mouse or any offspring of crosses with this strain should be mindful of the genetic impact that may be playing a role.

## Implications

In addition, research should explore whether mice with solely amyloid mutations exhibit similar disruptions in wheel-running as the dual Tgs examined here. This should be examined as a function of age and zinc administration. Zinc intake can also disturb the balance of other biometals, including copper. Excess Zn can lead to deficiencies in Cu (Maret and Sandstead, 2006). Rats given 10 ppm Zn had spatial memory deficits; however, when also given 0.025 ppm Cu, these spatial memory deficits were remediated (Chrosniak et al., 2006; Railey et al., 2010, 2011). Thus the effect of Zn plus Cu on circadian activity should also be examined.

## Conclusion

In summary, we have shown that the dual Tg mouse model of AD has alterations in wheel-running behavior that is impacted by Zn administration. Dual Tg mice showed lower activity compared to other genotype groups, particularly as a function of age, and Zn led to a significant reduction in their wheel-running activity.

## Data Availability Statement

The raw data supporting the conclusion of this article will be made available by the authors, without undue reservation.

## Ethics Statement

The animal study was reviewed and approved by the George Mason University IACUC.

## Author Contributions

SL and JF wrote and revised the manuscript. PK conducted the statistical analyses and provided plots and captions of the all data. MS conducted the wheel-running experiments and gathered all data used in this manuscript. All authors contributed to the article and approved the submitted version.

## Conflict of Interest

The authors declare that the research was conducted in the absence of any commercial or financial relationships that could be construed as a potential conflict of interest.
